# Purified Bighead protein efficiently promotes head development in the South African clawed frog, *Xenopus laevis*

**DOI:** 10.17912/micropub.biology.000347

**Published:** 2021-01-05

**Authors:** Gabriele Colozza

**Affiliations:** 1 IMBA - Institute of Molecular Biotechnology

## Abstract

Vertebrate embryonic development is regulated by a few families of extracellular signaling molecules.* Xenopus laevis *embryos offer an excellent system to study the cell-cell communication signals that govern embryonic patterning. In the frog embryos, Wnt/β-catenin plays a pivotal role in regulating embryonic axis development, and modulation of the Wnt pathway is required for proper antero-posterior patterning. Recently, a novel secreted, organizer-specific Wnt inhibitor, Bighead, was identified that acts by downregulating Lrp6 plasma membrane levels. Here, I describe a method to purify biologically active Bighead protein and confirm that Bighead promotes *Xenopus* head development*.*

**Figure 1. Purified Bighead Strep-Tag induces Lrp6 relocalization and promotes expansion of anterior tissues in Xenopus laevis:**
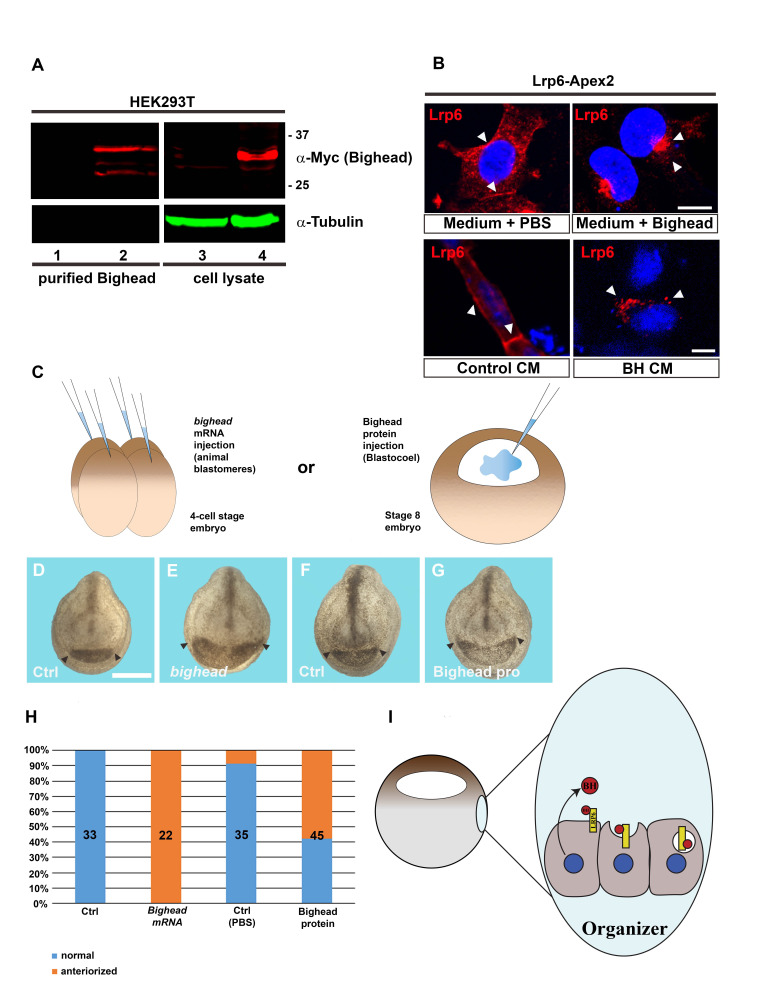
**A)** Western blot confirming Bighead-Myc Streptag purification from extracellular medium. HEK-293T cells were transfected with Bighead-Myc Streptag plasmid. Conditioned medium containing Bighead Streptag was collected from transfected cells and affinity purified by using streptactin columns. Purification of secreted Bighead protein was assessed using an anti-Myc antibody. Bighead was detected only in transfected cells (lanes 2 and 4) and was absent in control cells (lanes 1 and 3). Note that two major bands were observed, between 25 and 37 kDa, both in whole cell lysate and after purification. These may reflect different post-translational modifications that affect protein molecular weight and, hence, electrophoretic migration.Tubulin, present just in the cell lysate, was used as a loading control. **B)** When applied to the culture medium of HEK-293T cells expressing a chimeric Lrp6-Flag Apex2 construct, purified Bighead-Myc Streptag induces Lrp6 relocalization in intracellular puncta, reminiscent of endosomal vesicles, as compared to control cells treated with PBS only. A similar effect is obtained when cells are incubated with conditioned medium obtained from Bighead expressing cells (BH CM). Note how Lrp6 changes its localization from the plasma membrane (cells treated with control media; see arrowheads) to intracellular vesicles that tend to concentrate in the nuclear bay (arrowheads). Scale bars represent 10 μm. **C)** Cartoon depicting embryo injections with *bighead-myc streptag* mRNA (left) or purified protein (right). **D-G)** Frontal view of embryos injected as shown in panel C, and collected at tailbud stage (st. 22). 200 ng of *bighead-myc streptag* mRNA were injected in each blastomere of *Xenopus* embryos at the 4-cell stage. Injected embryos show clear expansion of anterior structures, such as the cement gland (E, black arrowheads pointing at cement gland borders), as compared to uninjected WT embryos (D). Embryos injected with purified Bighead protein inside the blastocoel cavity at stage 8 (G), displayed head enlargement compared to PBS-injected controls (F), akin to mRNA injections. Scale bar in D represents 500 μm. All embryos were photographed at the same magnification. **H)** Quantifications of the experiment shown in D-G. Plotted are the percentages of embryos showing anteriorization phenotype (evaluated by the enlargement of head and cement gland). The total number (n) of embryos used for this experiment is shown in each column. **I)** Cartoon showing the proposed model for Bighead activity during patterning of the *Xenopus* gastrula. *In vivo*, Bighead is secreted by the Spemann organizer, where it represses Wnt signaling by binding to Lrp6 receptor and inducing its internalization.

## Description

Previous RNA-sequencing experiments were performed on *Xenopus* *laevis* embryos to reveal novel Spemann organizer-specific genes. Analysis on the dorsal and ventral transcriptomes of early frog gastrula revealed an “organizer signature” of genes dorsally expressed and under the control of maternal Wnt signaling (Ding *et al.*, 2017a and b). Among the new genes found in the dorsal side of *Xenopus*, we identified an additional member of the Dapper antagonist of β-catenin (Dact) family of proteins, called *dact4* (Colozza and De Robertis, 2020) as well as several secreted Wnt antagonists, including *vlk* (Vertebrate lonesome kinase, also known as Pkdcc) (Ding *et al.*, 2017a) *angiopoietin-like 4* (Kirsch *et al.*, 2017) and *bighead* (Ding *et al.*, 2018). The latter represents the only member of a new family of secreted factors conserved in frogs and fishes, but absent in higher vertebrates such as mammals (Ding *et al.*, 2018). Interestingly, protein structure prediction indicated relevant homology with the prodomain region of the latent form of myostatin/growth and differentiation factor 8 (GDF8) (Ding *et al.*, 2018). *Xenopus* *bighead* (gene ID 108715768) encodes a Wnt antagonist that binds to the Lrp6 co-receptor and induces its internalization and degradation into lysosomes, downregulating canonical Wnt/β-catenin signaling. Overexpression of *bighead* mRNA into *Xenopus* embryos promotes the enlargement of head, eyes and cement gland, accompanied by expanded anterior neural markers, such as *rx2a*, *foxg1* and *otx2* (Ding *et al.*, 2018). On the other hand, morpholino-mediated knock-down of *bighead* has the opposite effect, as shown by the strong reduction in head development and expression of markers such as *otx2*. Altogether, these evidences suggest that *bighead* is required for head development in *Xenopus* embryos via inhibition of Wnt signaling (Ding *et al.*, 2018). Here, I show that secreted Bighead protein purified from the extracellular medium maintains its activity, as assessed in cell culture and *Xenopus* embryos. To this aim, I used a Streptag/Streptactin-based purification system, which harness the ability of a short tag (8 amino acid long) to bind strongly to modified streptavidin molecules (Maier *et al.*, 1998; Schmidt *et al.*, 1996; Voss and Skerra, 1997). Streptag was introduced at the C-term end of Bighead, where modifications are well tolerated and do not interfere with its signaling activity (Ding *et al.*, 2018). The Streptag was also preceded by a Myc tag, to allow for easier detection of Bighead protein. pCS2 plasmid containing recombinant *bighead-Myc-Streptag* DNA was transfected into HEK-293T cells. Conditioned medium (CM) containing Bighead protein was collected 48 hours after transfection, and applied to Streptactin columns for affinity purification. Media from empty pCS2 transfected cells was used as a control. Eluted protein was then dialyzed and concentrated in PBS using Millipore concentrators. Western blot analysis confirmed expression and purification of Bighead protein **(Fig. 1A)**. Notably, two major bands were observed between 25 and 37 kDa, suggesting possible post-translational modifications.200 ng of protein were then applied to the culture medium of HEK-293T cells expressing Lrp6-Flag-APEX2 (Colozza *et al.*, 2020). Compared to control, Lrp6-Flag-APEX2 relocated in intracellular clusters resembling endosomal vesicles, within 3 hours after treatment with purified Bighead protein **(Fig. 1B)**. Interestingly, both Bighead CM and purified protein showed similar effect on Lrp6 subcellular localization, supporting the specificity of Bighead activity. Then, I turned to *Xenopus* embryos to further characterize the activity of Bighead-Streptag **(Fig. 1C)**. mRNA injections induced strong enlargement of the head and cement gland **(Fig. 1D, E)**, a phenotype that is associated with inhibition of zygotic Wnt signaling (Niehrs, 2001; Ding *et al.*, 2018). Interestingly, injection of the purified protein into the blastocoel (a cavity that collects extracellular fluids and growth factors and plays important role in embryonic patterning) of stage 8 blastula embryos produced similar effects **(Fig. 1F-H)**. This result confirms that purified Bighead protein is active by operating at the extracellular level, as expected for a secreted factor, and promotes head enlargement in *Xenopus laevis*. This also suggests a possible mechanism for Bighead function in frog embryos, where extracellular Bighead protein (produced and secreted by cells in the Spemann organizer) attenuate Wnt signaling by inducing Lrp6 endocytosis, as proposed in the model in **Fig. 1I**.

In conclusion, this study provides a straightforward and user-friendly method to purify extracellular soluble factors that can be applied directly to cultured cells and vertebrate embryos to study cell-cell signaling modulation.

## Methods

**Cloning and mRNA transcription.** *Xenopus* *laevis bighead* cDNA was subcloned into a Gateway-adapted pCS2 contaning Strep-Tag (for C-terminal tagging). pCS2-Bighead Step-Tag was digested with NotI restriction enzyme. 1 μg of the linearized plasmid was used as a template for mRNA *in vitro* transcription, using the Sp6 mMessage mMachine and following manufacturer’s instructions.

***Xenopus* husbandry and embryo injection.** All animal experiments were performed in accordance to guidelines for animal welfare. *X. laevis* frogs were purchased from the Nasco Company. A sperm suspension was obtained from testicles manually dissected from male frogs, and crushed in 1 ml of 1x Marc’s Modified Ringers (MMR, 0.1 M NaCl, 2.0 mM KCl, 1 mM MgSO4, 2 mM CaCl 2,5 mM HEPES, pH7.4). Ovulation of female frogs was induced the night before experimentation by injecting 800 Units of human chorionic gonadotropin (hCG). The day after, frogs were let to spontaneously lay eggs in a high-salt solution (1.2 x MMR). Laid eggs were collected and fertilized with 200–300 μl of the sperm suspension. To remove the jelly-coat, fertilized eggs were treated with a 2% cysteine in 0.1x MMR solution pH 7.8, for about 7 min at room temperature (RT). Dejellied embryos were then cultured in 0.1x Marc’s modified Ringer’s solution and staged according to Nieuwkoop and Faber (1967). For mRNA injection, 200 ng of Bighead Strep-Tag mRNA were injected animally in each blastomere of 4-cell stage embryos. For protein injection, *Xenopus* embryos at blastula stage were injected into the blastocoele cavity with 40 nl Bighead Strep-Tag protein solution (250 ng/ml, in PBS). Embryos were then collected at stage 22-24 and analyzed for phenotype.

**Bighead protein expression and purification.** pCS2 Bighead Step-Tag was transfected into HEK 293T cells using Lipofectamine 2000 and following manufacturer’s instructions. 16 hours after transfection culture medium was replaced with fresh one, and cells were cultured for further 96 hours. The conditioned media from 5 transfected 10 cm-plates was collected and run through Strep-Tactin columns. Columns were washed and Bighead Strep-Tag protein was eluted following manufacturer’s instructions. Eluted protein was concentrated using Amicon concentrators (Merck Millipore), exchanging the eluent with PBS. Bighead protein was quantified using a BCA assay kit and a BSA (bovine serum albumin) standard curve. Medium from empty vector transfected cells was used as a negative control, and processed as described above.

## Reagents

– Lipofectamine 2000 (Thermo Fisher Sci., 11668030)

– Strep-Tag/Strep-Tactin purification kit (IBA Lifesciences)

– Flag antibody, mouse monoclonal (Sigma, M2 F1804); RRID:AB_262044

– anti-Myc antibody, rabbit polyclonal (GeneTex, GTX103436); RRID:AB_11162914

– anti alpha-Tubulin antibody, mouse monoclonal (Santa Cruz Biotech, sc-32293); RRID:AB_628412

– secondary antibodies for immunofluorescence: Cy3 donkey anti-mouse (Jackson ImmunoResearch, 715-165-150); RRID:AB_2340813

– secondary antibodies for infrared Western Blot: IRDye 680LT donkey anti-rabbit IgG (Li-Cor, 926-68023; RRID:AB_10706167); IRDye 800CW donkey anti-mouse 800 (Li-Cor, 926-32212; RRID:AB_621847)

– precast gels for SDS-PAGE, Novex WedgeWell 4-20%, Tris-Glycine, 1.0 mm, Mini Protein Gel, 10-well (Thermo Fisher Sci., XP04200BOX)

– mMessage mMachine, Sp6 in vitro transcription kit (Thermo Fisher Sci., AM1340)
